# Incongruence between morphotypes and genetically delimited species in the coral genus *Stylophora*: phenotypic plasticity, morphological convergence, morphological stasis or interspecific hybridization?

**DOI:** 10.1186/1472-6785-11-22

**Published:** 2011-10-04

**Authors:** Jean-François Flot, Jean Blanchot, Loïc Charpy, Corinne Cruaud, Wilfredo Y Licuanan, Yoshikatsu Nakano, Claude Payri, Simon Tillier

**Affiliations:** 1 Courant Research Center "Geobiology", University of Göttingen, Goldschmidtstraße 3, 37077 Göttingen, Germany; 2 CEA-Institut de Génomique, GENOSCOPE, Centre National de Séquençage, 2 rue Gaston Crémieux, CP5706, 91057 Evry Cedex, France; 3 UMR UPMC-CNRS-MNHN-IRD 7138, Département Systématique et Évolution, Muséum National d'Histoire Naturelle, Case Postale 26, 57 rue Cuvier, 75231 Paris Cedex 05, France; 4 URBO, Department of Biology, University of Namur, Rue de Bruxelle 61, 5000 Namur, Belgium; 5 UMR LOBP, Centre d'Océanologie de Marseille, Campus de Luminy, Case 901, 13288 Marseille Cedex 09, France; 6 UMR LOBP, Centre IRD de Tahiti, BP 529, 98713 Papeete, French Polynesia; 7 Br. Alfred Shields FSC Marine Station and Biology Department, De La Salle University, Manila 1004, Philippines; 8 Sesoko Station, Tropical Biosphere Research Center, University of the Ryukyus, Okinawa 3422, Japan; 9 UR COREUS, IRD, B.P. A5, 98848 Nouméa, New Caledonia

## Abstract

**Background:**

Morphological data suggest that, unlike most other groups of marine organisms, scleractinian corals of the genus *Stylophora *are more diverse in the western Indian Ocean and in the Red Sea than in the central Indo-Pacific. However, the morphology of corals is often a poor predictor of their actual biodiversity: hence, we conducted a genetic survey of *Stylophora *corals collected in Madagascar, Okinawa, the Philippines and New Caledonia in an attempt to find out the true number of species in these various locations.

**Results:**

A molecular phylogenetic analysis of the mitochondrial ORF and putative control region concurs with a haploweb analysis of nuclear ITS2 sequences in delimiting three species among our dataset: species A and B are found in Madagascar whereas species C occurs in Okinawa, the Philippines and New Caledonia. Comparison of ITS1 sequences from these three species with data available online suggests that species C is also found on the Great Barrier Reef, in Malaysia, in the South China Sea and in Taiwan, and that a distinct species D occurs in the Red Sea. Shallow-water morphs of species A correspond to the morphological description of *Stylophora madagascarensis*, species B presents the morphology of *Stylophora mordax*, whereas species C comprises various morphotypes including *Stylophora pistillata *and *Stylophora mordax*.

**Conclusions:**

Genetic analysis of the coral genus *Stylophora *reveals species boundaries that are not congruent with morphological traits. Of the four hypotheses that may explain such discrepancy (phenotypic plasticity, morphological stasis, morphological convergence, and interspecific hybridization), the first two appear likely to play a role but the fourth one is rejected since mitochondrial and nuclear markers yield congruent species delimitations. The position of the root in our molecular phylogenies suggests that the center of origin of *Stylophora* is located in the western Indian Ocean, which probably explains why this genus presents a higher biodiversity in the westernmost part of its area of distribution than in the "Coral Triangle".

## Background

The reason why most marine life forms, including corals, display their peak of biodiversity in the so called 'Coral Triangle' in Southeast Asia remains mysterious and much debated [1,2]. The rare examples of sea creatures that do not conform to this general pattern may offer information crucial for our understanding of its root causes, provided that a solid taxonomic framework is available to interpret their present and past distribution (which is unfortunately rarely the case). Several such exceptions to the 'Coral Triangle' rule can be found in the scleractinian coral family Pocilloporidae that comprises the three genera *Pocillopora*, *Seriatopora *and *Stylophora*: although morphospecies of *Seriatopora *are most diverse in the Coral Triangle and therefore seem to follow the rule, *Pocillopora *"has what appears to be many regional endemics, especially in the central and far eastern Pacific" and *Stylophora *"has a higher diversity in the western Indian Ocean and Red Sea than in the central Indo-Pacific" [3]. However, ongoing genetic studies of species boundaries in *Pocillopora *and *Seriatopora *suggest that, even though morphological descriptions of pocilloporid corals appear well founded in some locations [4-6], in others places current taxonomy is a poor predictor of the actual number of species [7-11].

**Figure 1:**
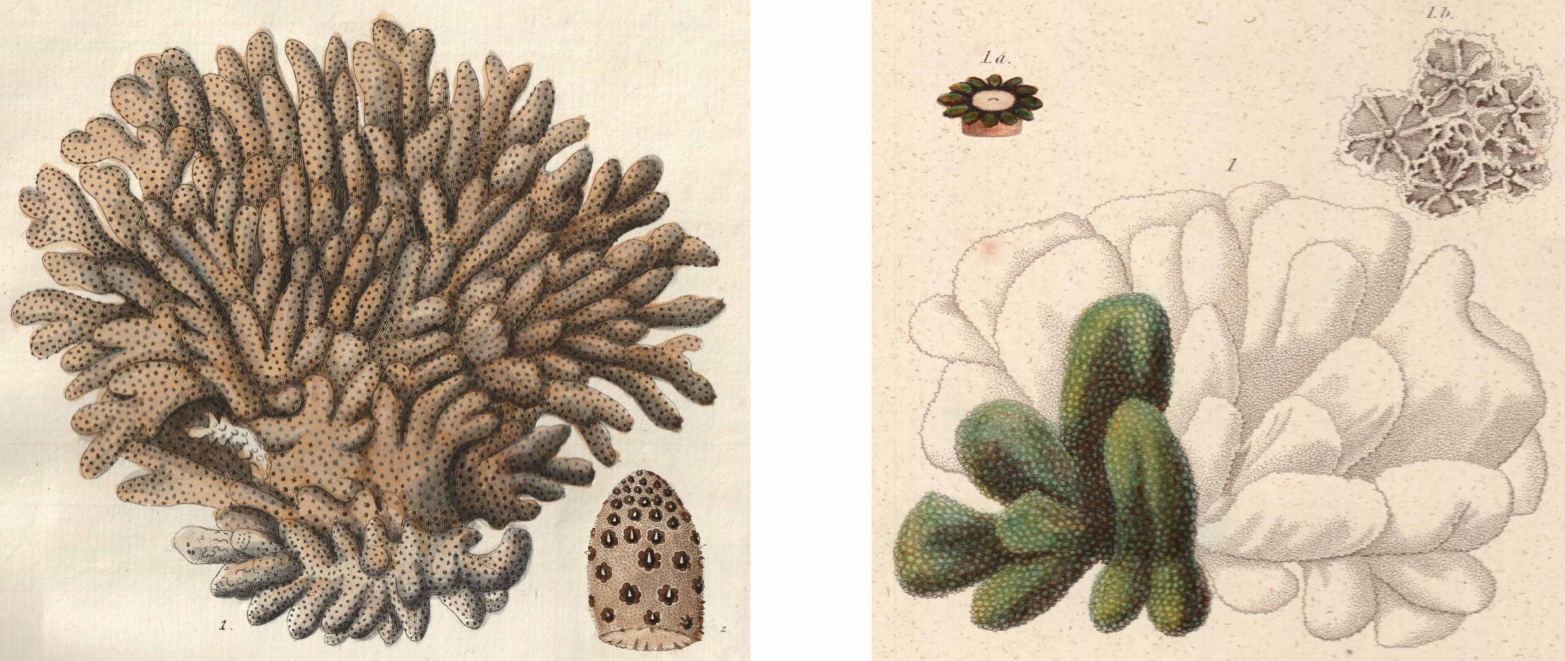
**Drawings of the type specimens of *S. pistillata *and *S. mordax***. The drawing of the type of *S. pistillata *(left) is from [25], the drawing of the type of *S. mordax *(right) is from [14].

Molecular studies of *Stylophora *have focused so far on a single species, the "lab rat" *Stylophora pistillata *(Esper, 1797), without delving into the delineation of interspecific boundaries [12,13]. *S. pistillata *was originally described "aus den ostindischen Meeren" [14] (from the East Indian seas, in the "Coral Triangle") as characterized by short twisted dichotomous branches ("Madrepora aggregata, ramulis conglomeratis brevibus, dichotomis; stellis crenatis profundis, pistillo in medio elongato erecto" [14]; Figure 1, left side), and is considered to occur over the entire Indo-Pacific (from the Red Sea to Polynesia). The distinction between *S. pistillata *and *Stylophora mordax *(Dana, 1846) has long been debated in the literature: *S. mordax *is considered by some authors as a junior synonym of *S. pistillata *[15,16,3] and by others as a distinct species [17-24]. *S. mordax *was originally described from Fiji as having "branches nearly simple, much compressed, not thinner at apex, scarcely flabellate, 1/2 to 1 inch broad, and 1/3 of an inch thick; polyps with a pale yellowish disk, and short tentacles of a bright green colour, deep brown at base. Corallum with the cells strongly vaulted, and the surface, therefore, decidedly scabrous" (Figure 1, right side) [25]; however, intermediary forms between *S. mordax *and *S. pistillata *are commonly found in the field, and the areas of distribution of these two morphotypes are nearly identical, further adding to the confusion.

Another widespread species according to Veron [3] is *Stylophora subseriata *(Ehrenberg, 1834), also found across the Indo-Pacific region, whereas the five other morphospecies of this genus are restricted to the Red Sea and the Gulf of Aden (*Stylophora kuehlmanni *Scheer and Pillai, 1983; *Stylophora danae *Milne Edwards and Haime, 1850; *Stylophora mamillata *Scheer and Pillai, 1983), to Madagascar (*Stylophora madagascarensis *Veron, 2000) or to both of these regions (*Stylophora wellsi *Scheer, 1964). Hence, the pattern of occurrence of the various species of *Stylophora *seems to contradict strongly the common "Coral Triangle" center of biodiversity model. However, recent reports of *S. danae *and *S. kuehlmanni *from the Philippines [26] have started to question this pattern, raising further concern that morphological species may not correspond to actual genetic entities (and indeed, Sheppard and Sheppard [27] considered *S. danae*, *S. kuehlmanni *and *S. subseriata *as ecomorphs of *S. pistillata*).

**Figure 2:**
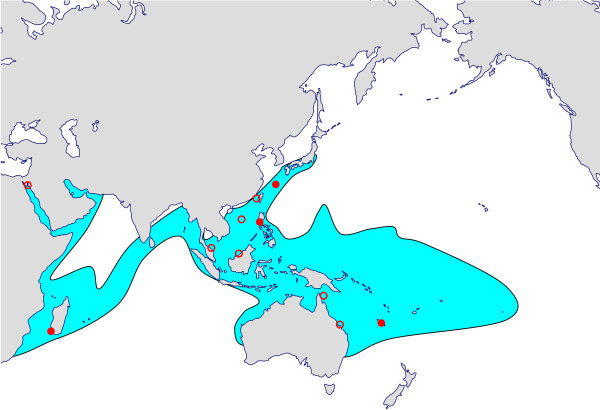
**Map of the area of distribution of the genus *Stylophora *showing our sampling locations**. Full circles show the locations where we collected samples, whereas empty circles mark locations where sequences were obtained from the literature. The area of distribution of the genus *Stylophora *(modified from [3] and [68]) is shown in blue (basemap from http://d-maps.com/).

Experimental work has shown that *S. pistillata *is phenotypically plastic [28,29] under the influence of environmental parameters such as gravity [30] and water flow [31]. Moreover, evidence of intergeneric hybridization between *Stylophora *and *Pocillopora *has been reported in the literature [32], suggesting that hybridization may *a fortiori *occur among various *Stylophora *species. Finally, morphological stasis and phenotypic convergence, albeit not reported yet in the genus *Stylophora*, may lead to further underestimation of the actual number of species in this genus by causing several genetically distinct species to be lumped under a single name. To find out whether phenotypic plasticity, interspecific hybridization, morphological stasis and phenotypic convergence obscure the taxonomy of *Stylophora*, we conducted a genetic analysis of 70 corals of this genus collected in Madagascar, Okinawa, the Philippines and New Caledonia; published sequences of *Stylophora *individuals from Australia, Malaysia, the South China Sea, Taiwan and Okinawa were also scrutinized (Figure 2). As a first step towards a future taxonomic revision of this genus, we report here the result of our attempt to determine the true number of *Stylophora *species occurring at these various locations and to test whether the unusual biogeographic pattern reported for this genus holds true.

## Results

### Phylogenetic analysis of mitochondrial sequences reveals the presence of two *Stylophora *clades in Madagascar vs. a single one in the Pacific Ocean

**Figure 3:**
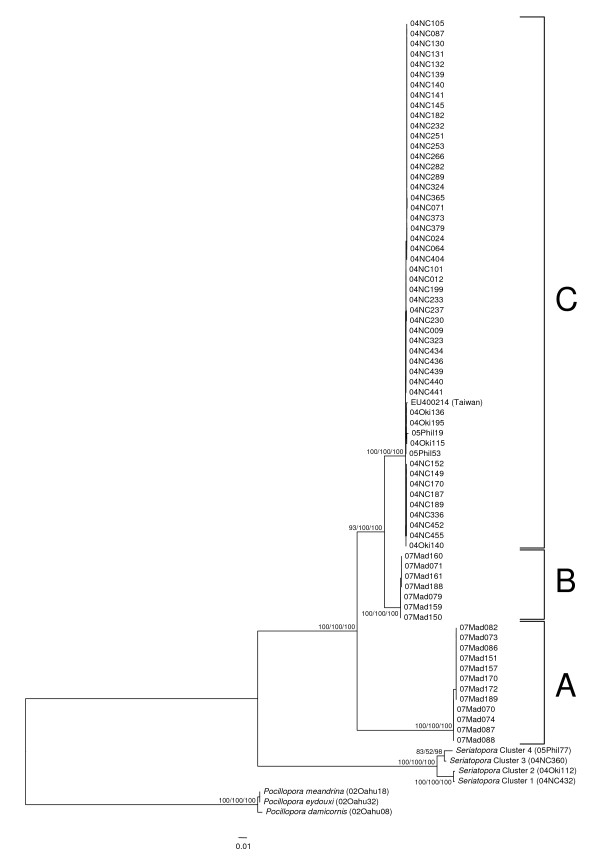
**Phylogenetic tree of mitochondrial DNA sequences (ORF and CR combined)**. Outgroup sequences are from [6] and [7], whereas one ingroup sequence comes from the complete mitochondrial genome of one *Stylophora pistillata *individual from the Penghu Islands near Taiwan [34]. This haplotree was generated with PhyML using the TPM3uf+G model suggested by jModelTest. Bootstrap values obtained using maximum likelihood, neighbor-joining and parsimony (1000 replicates each) are displayed next to each node. The three main clades are delineated with brackets and labeled A, B and C.

We successfully sequenced from all individuals sampled a portion of the mitochondrial ORF and the putative control region (CR), two markers that had previously been characterised as highly variable in the closely related genus *Pocillopora *[33] (but see [34,5] for a different interpretation of these regions). Since these markers gave results that were completely congruent, only the result of their combined analysis is presented here (Figure 3). The reciprocal monophyly of each pocilloporid genus received very strong bootstrap support (100% using maximum likelihood, neighbor-joining and parsimony), whereas three clades of *Stylophora *emerged from the phylogenetic analysis: clade A comprised 12 individuals, all from Madagascar, clade B comprised 7 individuals, also all from Madagascar, whereas clade C comprised the remaining 51 individuals from New Caledonia, Okinawa and the Philippines plus one previously published sequence (from the complete mitochondrial genome of one *Stylophora pistillata *individual from Taiwan [34]). All three clades were very strongly supported (100% bootstrap support using all three methods).

### Haploweb analysis of nuclear ITS2 sequences shows that these three clades represent distinct species

We obtained nuclear internal transcribed spacer 2 (ITS2) sequences from all individuals sampled, and analyzed them together with the single published *Stylophora *ITS2 sequence available from GenBank (also from a *Stylophora pistillata *individual from Taiwan [35]). Despite its multiple-copy nature and its concerted mode of evolution [36], the ITS2 behaved in the present study just like a "regular" single-copy nuclear marker, with each individual harboring either one or two sequence types. Moreover, most ITS2 sequences types found co-occurring in some individuals were also observed occurring alone in other coral colonies, suggesting that these sequence types were allelic and segregated in a Mendelian fashion: for this reason we decided to call "heterozygotes" all individuals found to possess two different ITS2 types, even though we could not be totally sure that all ITS2 sequences obtained were really allelic (in the closely related genus *Pocillopora*, for instance, three ITS2 sequences types were observed in one individual [6]).

**Figure 4:**
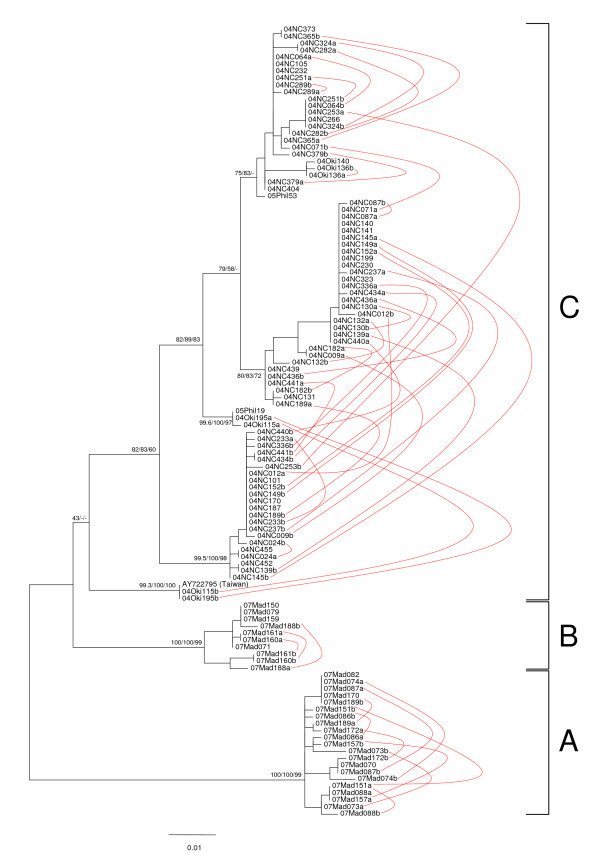
**Haploweb of ITS2 sequences**. This graph was derived from a phylogenetic tree (obtained with PhyML using the TIM2+G model suggested by jModelTest) by drawing curves connecting haplotypes found co-occurring in heterozygous individuals [9]. The sequence of one *Stylophora pistillata *individual from the Penghu Islands near Taiwan [35] is included in the figure, and bootstrap values obtained using maximum likelihood, neighbor-joining and parsimony (1000 replicates each) are displayed next to each node. The position of the root was inferred from the mitochondrial DNA phylogeny (Figure 3). This approach delineated three reproductively isolated pools of *Stylophora *sequences, labeled A, B and C as each of these groups corresponded to one of the mitochondrial clades of Figure 3.

Molecular phylogenetic analyses revealed many clades of sequences, several of which were very strongly supported (Figure 4). In order to determine which of these clades were conspecific and which ones belonged to different species, curves were added connecting sequences found co-occurring in heterozygous individuals, thereby converting the ITS2 tree into a haploweb [9]. This approach revealed three distinct pools of ITS2 sequences, corresponding to the three clades obtained from mitochondrial DNA. The monophyly of clades A and B in our ITS2 dataset received very strong bootstrap support using all three methods, whereas the monophyly of clade C was only weakly supported using maximum likelihood (<50% bootstrap support) and not at all supported using neighbor-joining and parsimony. None of the 43 heterozygous individuals in our dataset contained ITS2 sequences from two different clades: therefore, clades A, B and C correspond to three distinct species according to the mutual allelic exclusivity criterion [37,9].

### Phylogenetic analysis of nuclear ITS1 sequences reveals the existence of a fourth species of *Stylophora *in the Red Sea

**Figure 5:**
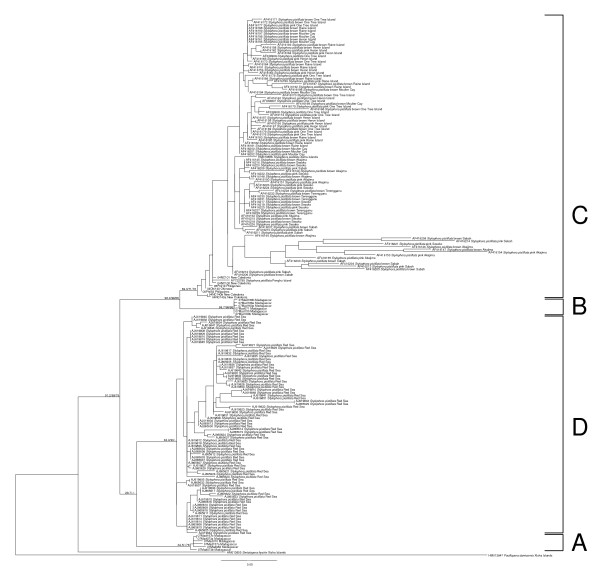
**ITS1 molecular phylogeny of the genus *Stylophora***. In addition to ITS1 sequences from selected individuals of species A, B and C, data from published articles [69,12,35,13] and from an unpublished study by Feng You and Hui Huang [GenBank: HM013847, HM013855, HM013856] were included in the haplotree (generated with PhyML using the TPM2+G model suggested by jModelTest).

Even though the ITS2 of scleractinian corals is generally easier to align and more informative than ITS1 [35] and is therefore more commonly used, the vast majority of ITS sequences available to date for the genus *Stylophora *are ITS1 sequences. In order to compare the results of our study with those previously published data, the ITS1 regions of 14 representative individuals from our molecularly defined species A, B and C were sequenced and aligned with sequences available in GenBank. Alignment was indeed markedly more uncertain for ITS1 than for ITS2, and the resulting alignment was rather short (345 positions including many gaps, vs. 700 positions for ITS2 and 2645 positions for the combined mitochondrial markers); nevertheless, the resulting phylogeny revealed interesting patterns and is therefore presented here (Figure 5).

The three species delimited from the mitochondrial and ITS2 datasets were recovered as distinct clades of ITS1 sequences: the monophyly of species B was very strongly supported (>98% bootstrap support using all three methods), whereas the monophyly of species C received weaker bootstrap support and the monophyly of species A was very weakly supported. All previously published ITS1 sequences from Australia, Malaysia, the South China Sea, Taiwan and Japan turned out to belong to species C, whereas all published *Stylophora *sequences from the Red Sea fell in a well supported fourth clade D (>90% bootstrap support using maximum likelihood and neighbor-joining) that can be considered a distinct species following the criterion of reciprocal monophyly.

## Discussion

### Haplowebs are useful tools to delineate species

The present study confirms the usefulness of our recently proposed haploweb approach to deal with sequences of nuclear markers [9]. Whereas the corresponding phylogenetic tree supported the delineation of clades A and B but revealed a large number of clades among our sequences from the Pacific Ocean, haploweb analysis (Figure 4) showed that these various Pacific Ocean clades are actually conspecific (since many heterozygous individuals harbor sequences from two different clades). The monophyly of species C was only very weakly supported in the maximum-likelihood phylogeny (with a bootstrap value of only 43%) and not at all supported using neighbor-joining and parsimony: therefore, this species would probably not have been detected in our ITS2 data if we had not taken into account the information provided by the co-occurrence of phylogenetically distant alleles in some individuals. In contrast, haploweb analysis delineated three groups of alleles among our ITS2 sequences, a result perfectly congruent with the mitochondrial phylogeny obtained from the same set of individuals.

Unlike in our previous article [9], the haploweb presented here was built on a tree rather than a network. Indeed, tree-based haplowebs are more straightforward to draw than their network-based counterparts, and are also more informative since they display the genotype of each sequenced individual. However, precisely due to their larger information content, tree-based haplowebs tend to become messy when dealing with large datasets and/or non-monophyletic species: in such cases, network-based haplowebs often turn out to be faster to draw and easier to interpret than tree-based ones.

### Are molecularly delimited species of *Stylophora *congruent with morphology?

**Figure 6:**
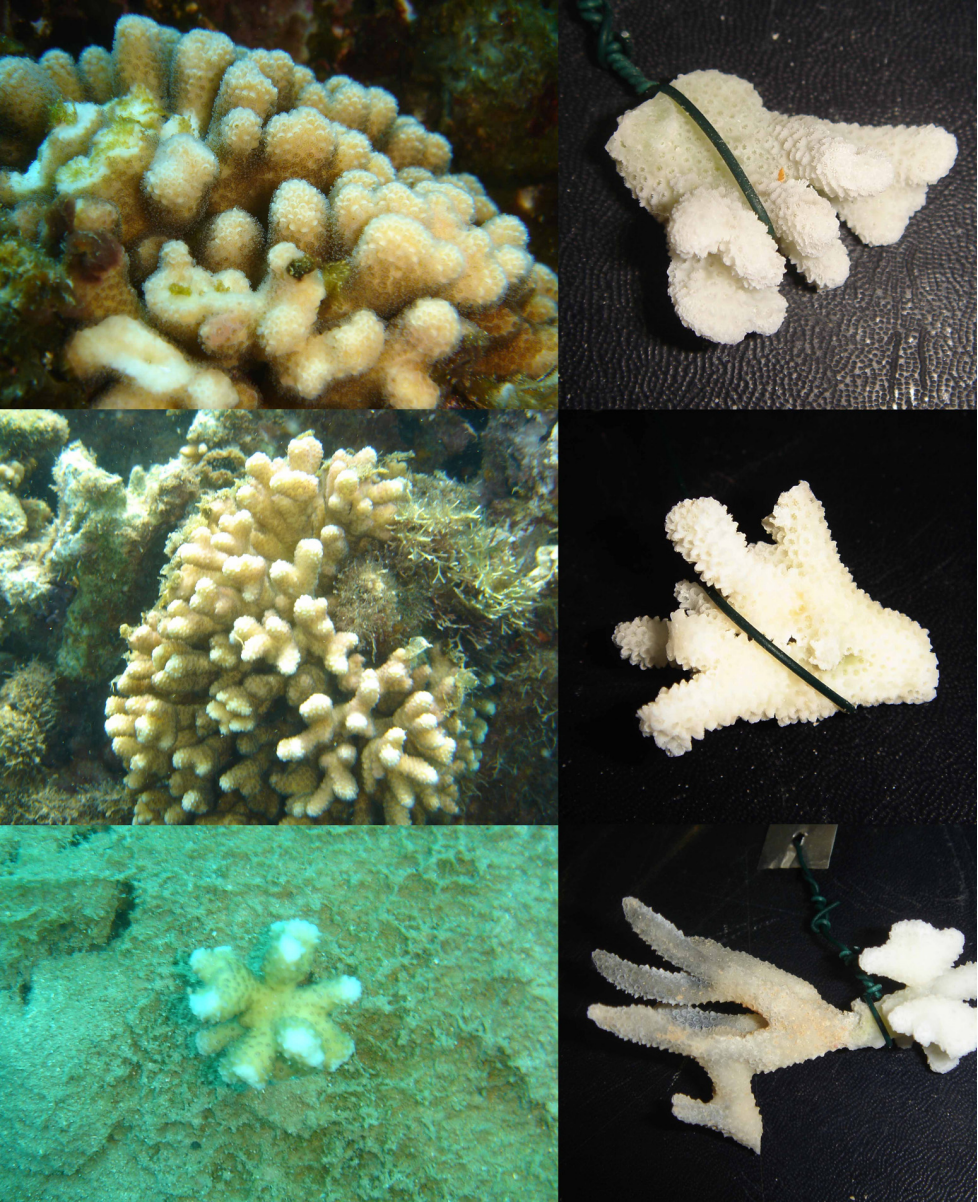
**Morphology of species A**. From top to bottom: colonies 07Mad074 sampled at 5 meters depth, colony 07Mad086 sampled at 6 meters depth and colony 07Mad170 sampled at 11 meters depth display the typical morphology of *S. madagascarensis *(thin, straight compact branches with small hoods). The green wire on the pictures of the skeletons has a diameter of 0.9 mm.

**Figure 7:**
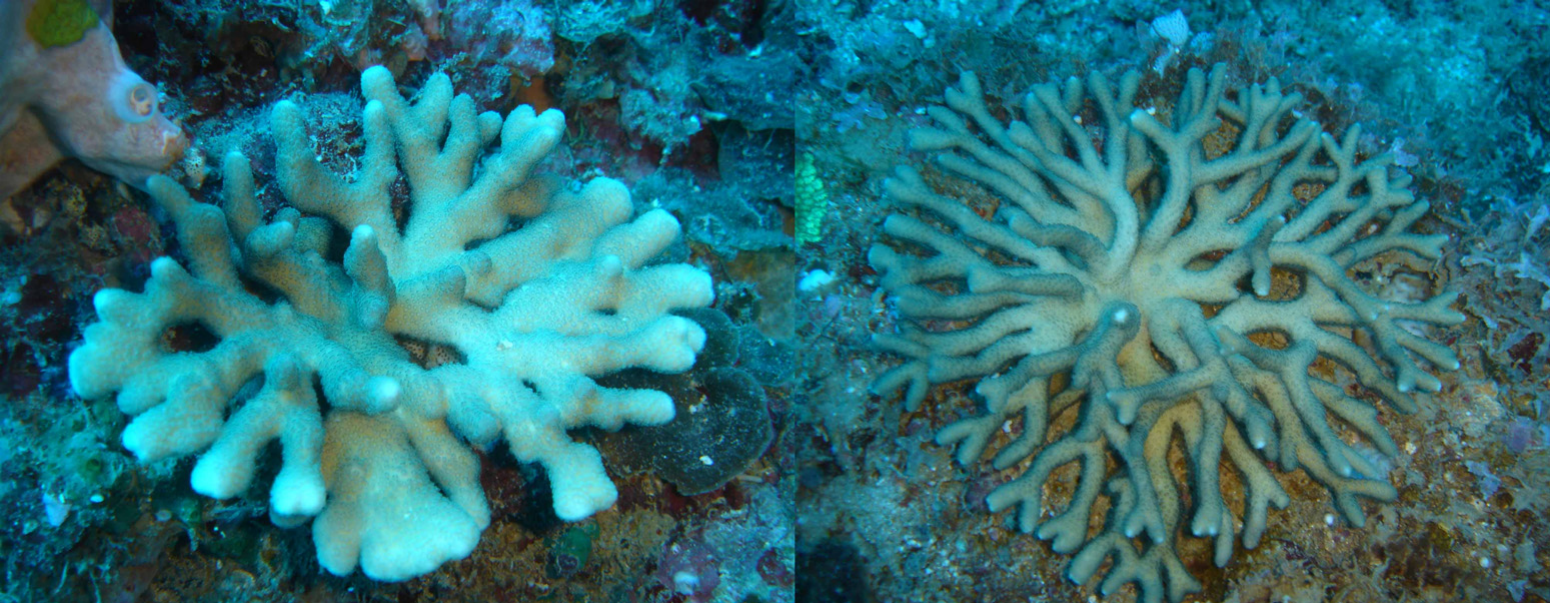
**Deep-water morphs of species A**. Colony 07Mad088 (left) and colony 07Mad097 (right) were both sampled at 30 meters depth and have thicker, less compact branches compared with typical *S. madagascarensis *morphology.

Most of the 12 individuals from species A displayed the characteristic morphology of *S. madagascarensis *(some examples are shown on Figure 6): "colonies have thin (up to 5 mm diameter), straight compact branches. Corallites are crowded and uniformly spaced on branch sides and ends. They have a slight development of hoods towards branch ends. They have small style-like columellae and six primary septa which are fused with the columellae. The coenosteum is covered with fine spicules. Tentacles are not extended during the day. Colour: Uniform tan, sometimes with pinkish branch bases." [38]. Since Veron's holotype was collected from the very same location in Madagascar where we conducted our sampling ("approximately 4 m depth, Tuléar, south-west Madagascar" [38]), there is little doubt that species A and *S. madagascarensis *are conspecific. According to Veron's description this species was "recorded only from shallow reef environments exposed to some wave action and, in south-west Madagascar, in shallow sheltered lagoons" [38]. In contrast, we found this species down to a depth of 30 meters, where it displayed less compact and thicker branches compared with the typical *S. madagacarensis *morphology (Figure 7): hence, *S. madagascarensis *appears to be more ecologically widespread and morphologically variable than previously thought.

**Figure 8:**
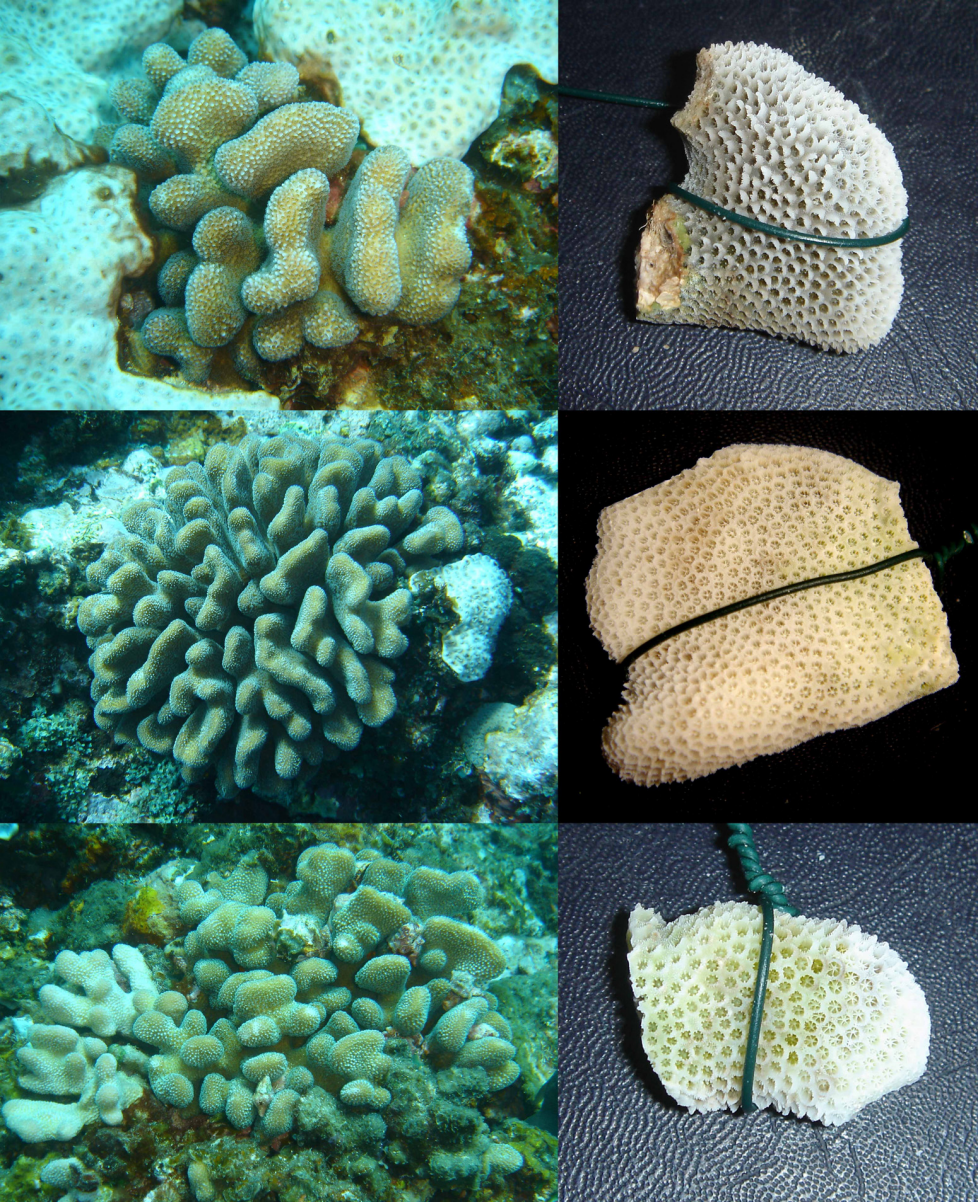
**Morphology of species B**. From top to bottom: colony 07Mad150 collected at 8 meters depth, colony 07Mad071 collected at 7 meters depth and colony 07Mad160 collected at 3 meters depth all display the typical morphology of *S. mordax *(short, compressed branches with well-developed hoods). The green wire on the pictures of the skeletons has a diameter of 0.9 mm.

All 7 individuals of species B from Madagascar had flattened stout branches (Figure 8). This corresponds unambiguously to the morphological description of *S. mordax*, but since *S. mordax *was originally described from Fiji in the Pacific Ocean it is dubious whether this name may be assigned to species B.

**Figure 9:**
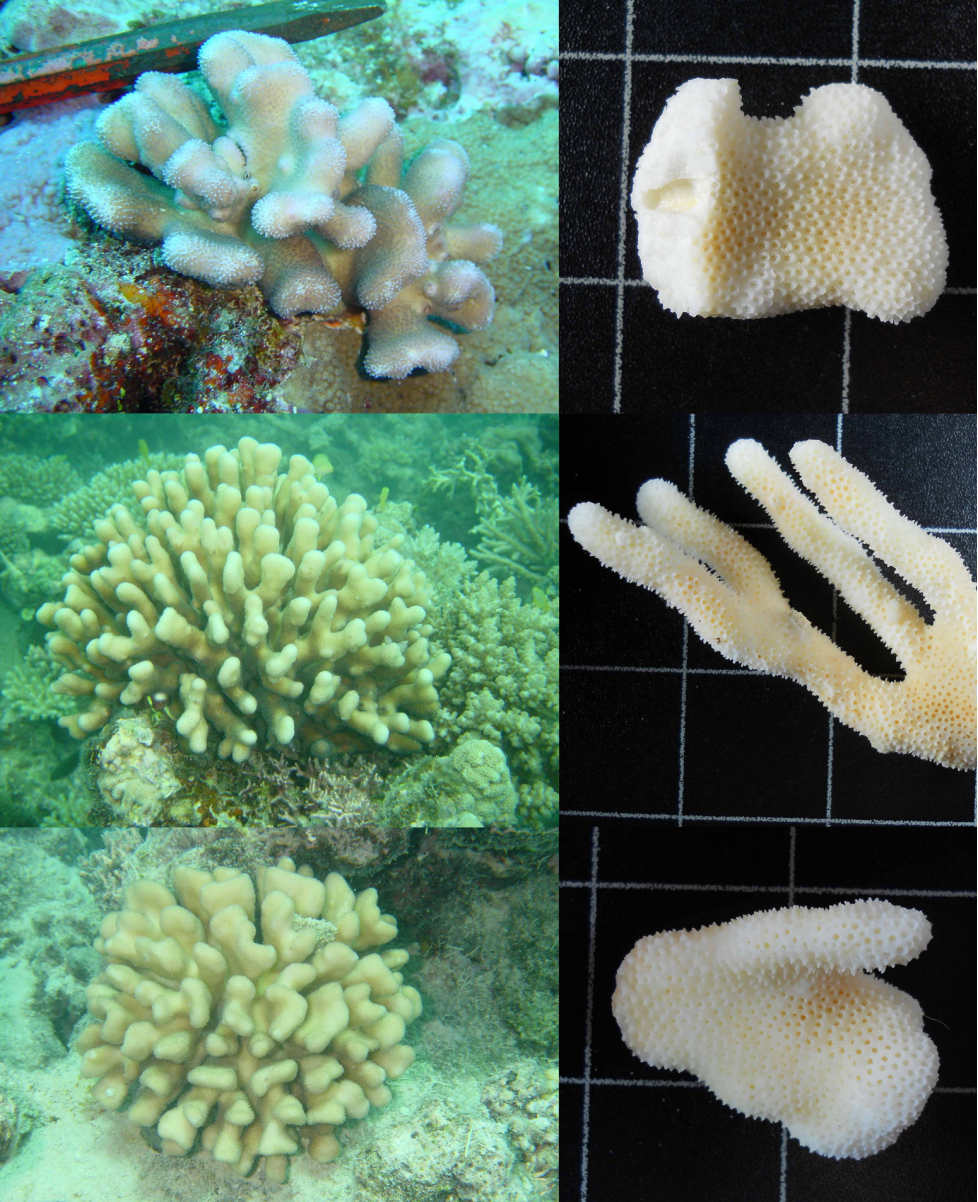
**Morphology of species C**. From top to bottom: colony 04NC253 was collected at 19 meters depth and displays the typical *S. mordax *morphology; colony 04NC323 was collected at 6 meters depth and displays the typical *S. pistillata *morphology; colony 04NC282 was collected at 6 meters depth and is intermediate in shape between *S. mordax *and *S. pistillata. *The distance between two white lines in the background is 30 mm.

**Figure 10:**
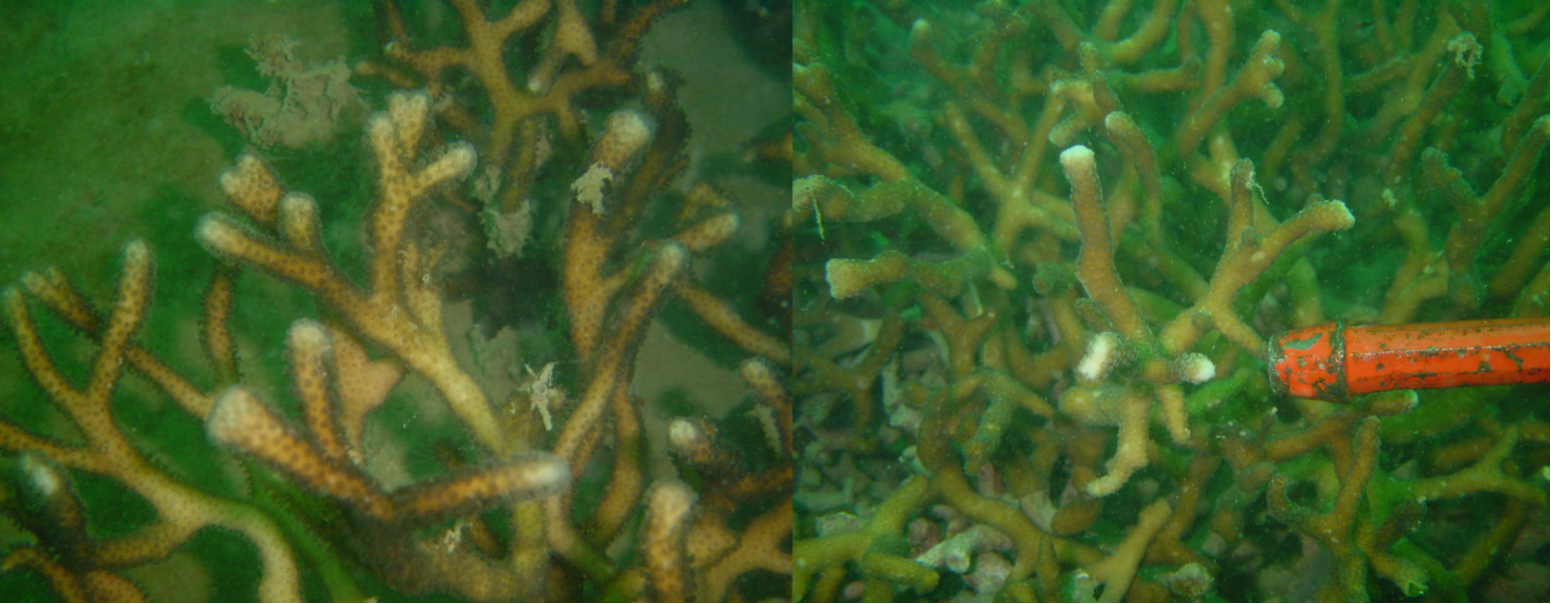
**Extremely elongated morph of species C from a very muddy site in New Caledonia**. Colonies 04NC149 (left) and 04NC170 (right) were sampled at 16 and 15 meters depth at the *Banc des Japonais *in New Caledonia.

Species C gathered all individuals collected from locations in the Pacific Ocean. It is characterized by extensive morphological variation, comprising individuals attributable to *S. pistillata *and others attributable to *S. mordax*, as well as morphotypes somehow intermediary in shape (Figure 9). Several colonies of this species collected in a very muddy location (*Banc des Japonais *in New Caledonia) displayed a peculiar phenotype characterized by extremely elongated thin branches (Figure 10). Published sequences of *S. pistillata *from Taiwan (ITS2, ORF) and from Okinawa, Taiwan, the South China Sea, Malaysia and Australia (ITS1) grouped with our sequences from species C, further supporting its putative identification as *S. pistillata*.

Finally, all published ITS1 sequences from the Red Sea fell in a distinct well-supported species D. The coral colonies sequenced were collected at depths of 2-4 meters in the Gulf of Aqaba and reported under the name *S. pistillata *[13], but according to another study the main morphospecies of *Stylophora *found in shallow waters in Aqaba is actually *S. mordax *[21]: therefore, species D probably comprises a mixture of *S. pistillata *and *S. mordax *morphotypes. Since the type localities of *S. pistillata *and *S. mordax *are both in the Pacific Ocean, another name will be required for species D (possibly *S. subseriata*, since this species was described from the Red Sea and was considered by some authors as a synonym of *S. pistillata *[27]).

### What causes the discrepancy between morphological and molecular species delimitations in *Stylophora*?

Our study revealed extensive morphological variation within species A and C: a more detailed genetic investigation using a larger number of variable markers such as microsatellites will be required to find out whether these variations are due to phenotypic plasticity or to underlying intraspecific genetic differences. However, phenotypic plasticity is well documented in *Stylophora *[30,31,28,29] and is therefore likely to be responsible for at least part of the observed morphological variations.

The occurrence of the *S. mordax *morphotype in both species B and C can hardly be explained by intra-specific variation alone, but may rather result from phenotypic convergence (whereby two non-sister species independently evolve similar morphologies) and/or morphological stasis (whereby the appearance of the common ancestor of two sister species is passed on to both of them). Since the sister-species relationship between B and C was very strongly supported by all molecular markers analyzed, morphological stasis appears more likely than phenotypic convergence to explain the similar appearance of species B and of some morphs of species C.

Even though the data available are not yet sufficient to pin down completely the causes of the interspecific morphological similarities and intraspecific phenotypic variations observed in *Stylophora*, the observed congruence between nuclear and mitochondrial phylogenies allows us to reject the hypothesis that hybridization could be responsible for the discrepancy between morphological and genetic species boundaries in this genus. This contrasts with previous reports that hybridization may be rampant in corals (e.g. [39-50]); instead, our results concur with two recent articles on the closely related genus *Pocillopora *[9,11] in suggesting that many such reports actually result from improper delineation of species boundaries and not from actual introgression between distinct genetic entities.

### A new light on the biogeography and biodiversity of *Stylophora *in the Indo-Pacific Ocean

Surprisingly, the incongruence between morphological species delimitations and genetic species boundaries revealed in our study does not seem to affect the general picture of the biogeographic distribution of *Stylophora *species: with a least three species in the westernmost part of its area of occurrence versus a single one so far in the "Coral Triangle", *Stylophora *stands confirmed as a blatant exception to the usual biodiversity pattern observed in tropical marine invertebrates. The mitochondrial and ITS2 phylogenies of *Stylophora *comprise only species A, B and C and do not contradict the topology of the more complete (but less resolved) ITS1 phylogeny: the sister-group relationship of species B and C is strongly supported by all markers, whereas the root of the mitochondrial and ITS1 phylogenies fall between species A (from Madagascar) and species B and C (respectively from Madagascar and from the Pacific Ocean). Even though the sister-group of species D could not be determined unambiguously due to the current lack of mitochondrial and ITS2 sequences for this species, the position of the root in our molecular phylogenies suggests that the center of origin of *Stylophora *is located in the western Indian Ocean. This hypothesis will need to be confirmed by analyzing more samples from key locations in the Red Sea, the Gulf of Aden and the Indian Ocean, but would explain well the unusual concentration of the biodiversity of this genus in the westernmost part of his area of distribution.

While waiting for a global taxonomic revision of the genus *Stylophora*, for the sake of taxonomic stability we recommend that the preliminary results presented here not be translated yet into nomenclature, but that each genetically delimited species be provisionally designated by a letter (i.e., "*Stylophora *sp. A", "*Stylophora *sp. B", "*Stylophora *sp. C" and "*Stylophora *sp. D"). It is only when a complete picture of the species boundaries of *Stylophora *over its whole area of distribution becomes available that names will be reliably assigned to each species: for instance, even though the name *S. madagascarensis *appears suitable for species A given its morphological traits and the location where it was collected, this species may very well have been described first under another name in a different location, in which case *S. madagascarensis *would become a junior synonym of the actual name of this species.

## Conclusions

Genetic analysis of the coral genus *Stylophora *reveals species boundaries that are not congruent with morphology. Of the four hypotheses capable of explaining such discrepancy (phenotypic plasticity, morphological stasis, morphological convergence, and interspecific hybridization), the first two seem likely to play a role but the fourth one is rejected since mitochondrial and nuclear markers yield congruent species delimitations. The center of origin of *Stylophora *appears to be located in the Indian Ocean, which probably explains why this genus presents a higher biodiversity in the westernmost part of its area of distribution than in the "Coral Triangle".

## Methods

### Sample collection and processing

Fragments from 70 *Stylophora *coral colonies were collected while scuba diving or snorkeling on reefs of New Caledonia, Okinawa (Japan), Bolinao (Philippines) and Toliara (Madagascar) between 2004 and 2007. Each colony sampled was photographed underwater and its depth recorded (Table 1). Coral tissues were preserved in a buffered guanidium thiocyanate solution [51,52] and their DNA purified on an ABI Prism 6100 Nucleic Acid PrepStation.

**Table 1:** Localization and depth of each *Stylophora *sample analyzed

Sample name	Coordinates	Depth (m)		Sample name	Coordinates	Depth (m)
04Oki115	(26°12'10"N, 127°19"12"E)	n.r.		04NC323	(20°42'39"S, 165°09'14"E)	6

04Oki136	(26°12'10"N, 127°19"12"E)	n.r.		04NC324	(20°42'39"S, 165°09'14"E)	6

04Oki140	(26°12'10"N, 127°19"12"E)	n.r.		04NC336	(20°35'42"S, 165°10'40"E)	26

04Oki195	(26°14'25"N, 127°27'50"E)	n.r.		04NC365	(20°40'12"S, 164°11'19"E)	22

04NC009	(22°26'54"S, 166°22'23"E)	2		04NC373	(20°40'12"S, 164°11'19"E)	15

04NC012	(22°26'54"S, 166°22'23"E)	6		04NC379	(20°40'12"S, 164°11'19"E)	14

04NC024	(22°26'54"S, 166°22'23"E)	1		04NC404	(20°40'20"S, 164°14'53"E)	4

04NC064	(22°38'30"S, 166°34'40"E)	16		04NC434	(20°39'58"S, 164°15'26"E)	25

04NC071	(22°40'54"S, 168°36'24"E)	1		04NC436	(20°41'39"S, 164°14'50"E)	24

04NC087	(22°37'20"S, 166°36'53"E)	5		04NC439	(20°41'39"S, 164°14'50"E)	23

04NC101	(22°37'20"S, 166°36'53"E)	9		04NC440	(20°41'39"S, 164°14'50"E)	23

04NC105	(22°01'01"S, 165°55'10"E)	26		04NC441	(20°41'39"S, 164°14'50"E)	21

04NC130	(22°22'59"S, 167°05'50"E)	1		04NC452	(20°41'39"S, 164°14'50"E)	1

04NC131	(22°22'59"S, 167°05'50"E)	1		04NC455	(20°41'39"S, 164°14'50"E)	1

04NC132	(22°22'59"S, 167°05'50"E)	1		05Phil19	(16°23'46"N, 119°54'03"E)	24

04NC139	(22°22'59"S, 167°05'50"E)	1		05Phil53	(16°26'22"N, 119°56'33"E)	13

04NC140	(22°22'59"S, 167°05'50"E)	1		07Mad070	(23°25'01"S, 43°38'36"E)	8

04NC141	(22°22'59"S, 167°05'50"E)	1		07Mad071	(23°25'01"S, 43°38'36"E)	7

04NC145	(22°22'59"S, 167°05'50"E)	1		07Mad073	(23°25'01"S, 43°38'36"E)	5

04NC149	(22°15'27"S, 166°24'33"E)	16		07Mad074	(23°25'01"S, 43°38'36"E)	5

04NC152	(22°15'27"S, 166°24'33"E)	16		07Mad079	(23°25'01"S, 43°38'36"E)	8

04NC170	(22°15'27"S, 166°24'33"E)	15		07Mad082	( 23°23'07"S, 43°38'18"E)	8

04NC182	(22°12'31"S, 166°24'55"E)	4		07Mad086	( 23°23'07"S, 43°38'18"E)	6

04NC187	(22°12'31"S, 166°24'55"E)	4		07Mad087	(23°30'20"S, 43°41'10"E)	30

04NC189	(22°12'31"S, 166°24'55"E)	4		07Mad088	(23°30'20"S, 43°41'10"E)	30

04NC199	(22°18'40"S, 166°27'26"E)	1		07Mad150	(23°23'29"S, 43°37'38"E)	8

04NC230	(20°46'18"S, 165°16'30"E)	14		07Mad151	(23°23'29"S, 43°37'38"E)	8

04NC232	(20°46'18"S, 165°16'30"E)	13		07Mad157	(23°23'29"S, 43°37'38"E)	7

04NC233	(20°46'18"S, 165°16'30"E)	9		07Mad159	(23°23'29"S, 43°37'38"E)	3

04NC237	(20°46'18"S, 165°16'30"E)	7		07Mad160	(23°23'29"S, 43°37'38"E)	3

04NC251	(20°34'59"S, 165°08'11"E)	21		07Mad161	(23°23'29"S, 43°37'38"E)	2

04NC253	(20°34'59"S, 165°08'11"E)	19		07Mad170	(23°22'58"S, 43°38'11"E)	11

04NC266	(20°34'59"S, 165°08'11"E)	7		07Mad172	(23°22'58"S, 43°38'11"E)	11

04NC282	(20°34'59"S, 165°08'11"E)	6		07Mad188	(23°22'58"S, 43°38'11"E)	5

04NC289	(20°34'59"S, 165°08'11"E)	2		07Mad189	(23°22'58"S, 43°38'11"E)	3

n.r. = not recorded

### PCR amplification and sequencing

Three DNA markers previously developed in the closely related genus *Pocillopora *[33] were amplified and sequenced for each individual: the nuclear ribosomal intergenic transcribed spacer 2 (ITS2), the mitochondrial ORF and the putative control region (CR). In addition, new primers were used to amplify the ITS1 region of a few selected individuals (Table 2). Amplifications were performed in 25 μl reaction mixes containing 1x Red Taq buffer, 264 μM dNTP, 5% DMSO, 0.3 μM PCR primers, 0.3 units Red Taq (Sigma), and 10-50 ng DNA. PCR conditions comprised an initial denaturation step of 60 s at 94°C, followed by 40-50 cycles (30 s denaturation at 94°C, 30 s annealing at 53°C, 75 s elongation at 72°C) and a final 5-min elongation step at 72°C. PCR products were sequenced in both directions with the same primers as for amplification, and chromatograms were assembled and cleaned using Sequencher 4 (Gene Codes).

**Table 2:** Primers used for DNA amplification and sequencing

Marker	Primer name	Primer sequence	Reference
ITS1 (nuclear)	F18S1	5'-CGATYGAAYGGTTTAGTGAGGC-3'	this study

ITS1 (nuclear)	ITSc1-3	5'- CATTTGCGTTCAAAGATTCG-3'	this study

ITS2 (nuclear)	ITSc2-5	5'-AGCCAGCTGCGATAAGTAGTG-3'	[4]

ITS2 (nuclear)	R28S1	5'-GCTGCAATCCCAAACAACCC-3'	[4]

ORF (mitochondrial)	FATP6.1	5'-TTTGGGSATTCGTTTAGCAG-3'	[6]

ORF (mitochondrial)	RORF	5'-SCCAATATGTTAAACASCATGTCA-3'	[6]

CR (mitochondrial)	FNAD5.2deg	5'-GCCYAGRGGTGTTGTTCAAT-3'	[6]

CR (mitochondrial)	RCOI3deg	5'-CGCAGAAAGCTCBARTCGTA-3'	[7]

### Determination of nuclear haplotypes

The ITS2 chromatogram pairs obtained from 43 individuals contained double peaks, indicating that each of these individuals harbored two sequence types. Finding out the sequence types was trivial for 5 individuals whose chromatograms contained only one double peak. Furthermore, 21 other chromatogram pairs had numerous double peaks, a situation typical of length-variant heterozygotes [53,54] that allowed direct deconvolution of their superposed sequences using the program CHAMPURU [55] (available online at http://www.mnhn.fr/jfflot/champuru). The remaining 17 chromatograms pairs had several double peaks (at most 9), as expected from heterozygotes with no intra-individual length variation: we first attempted to resolve their haplotypes statistically by reference to the rest of the dataset using SeqPHASE [56] (available online at http://www.mnhn.fr/jfflot/seqphase) and PHASE [57], but only 7 individuals were phased unambiguously, i.e., with posterior probabilities equal or nearly equal to 1 (04NC064, 04NC182, 04NC251, 04NC282, 04NC324, 04NC436, 07Mad087). Among the 10 remaining heterozygotes, the haplotypes of 2 individuals (07Mad151, 07Mad189) were deduced directly from their chromatograms thanks to clear-cut differences in peak sizes (reflecting either differences in copy number in ribosomal DNA arrays or differential amplification during PCR), and the sequences of the 8 others (04NC024, 04NC132, 04NC365, 04NC379, 07Mad073, 07Mad074, 07Mad088, 07Mad157) were inferred using Clark's method [58]. Length-variant heterozygosity was also observed in the ITS1 chromatograms of five individuals, all of which were resolved using CHAMPURU.

### Phylogenetic analyses and haploweb construction

All haplotype sequences were deposited in public databases [GenBank:JN558840-JN559111]. ORF sequences were aligned in MEGA5 [59] by taking advantage of the high degree of conservation of their aminoacid translations: all sequences from *Stylophora *were first aligned by hand as there were only few indels, before aligning them with outgroup sequences from *Pocillopora *and *Seriatopora *using the MEGA5 implementation of MUSCLE [60]. CR, ITS1 and ITS2 sequences were aligned using MAFFT's Q-INS-I option [61,62]. Since the two mitochondrial markers ORF and CR yielded congruent phylogenies, they were concatenated and only the result of the combined analysis is presented here. The best suited nucleotide model among 88 possible ones was determined for each dataset following the Bayesian Information Criterion [63] as implemented in jModelTest [64], and used to perform maximum-likelihood phylogenetic analyses in PhyML [65] with 1000 bootstrap replicates [66]. Additional bootstrap analyses (1000 replicates) using neighbor-joining (K2P model, pairwise deletion) and parsimony (dataset collapsed using FaBox [67], complete deletion) were conducted in MEGA5. The Newick format haplotype trees ("haplotrees") produced by PhyML were converted into enhanced metafiles (emf) using the program FigTree 1.3.1 (available online at http://tree.bio.ed.ac.uk/software/figtree/), then imported in Microsoft PowerPoint. The ITS2 haploweb was obtained from the corresponding haplotree by drawing connections between haplotypes found co-occurring in heterozygous individuals [9].

## Authors' contributions

JFF carried out fieldwork, DNA extractions and PCR amplifications, analyzed the results and drafted the manuscript. CC sequenced all PCR products. JB, LC, WL, YN and CP provided logistic support, participated in fieldwork and revised the manuscript. ST supervised the study and revised the manuscript, the final version of which was read and approved by all authors.
